# Detection of ivermectin and fipronil resistance in *Rhipicephalus sanguineus* sensu lato in Maha Sarakham, Thailand

**DOI:** 10.14202/vetworld.2023.1661-1666

**Published:** 2023-08-19

**Authors:** Bunnada Siriporn, Amornrat Juasook, Nattarika Neelapaijit, Piyatida Kaewta, Zhiliang Wu

**Affiliations:** 1 Veterinary Infectious Disease Research Unit, Faculty of Veterinary Sciences, Mahasarakham University, Maha Sarakham, Thailand; 2 Bioveterinary Research Unit, Faculty of Veterinary Sciences, Mahasarakham University, Maha Sarakham, Thailand; 3 Bunsiri Animal Hospital, Samut Prakan, Thailand; 4 Faculty of Veterinary Sciences, Mahasarakham University, Maha Sarakham, Thailand; 5 Department of Parasitology and Infectious Disease, Graduate School of Medicine, Gifu University, Gifu, Japan

**Keywords:** bioassay, dog, fipronil, ivermectin, resistance, *Rhipicephalus sanguineus* sensu lato, Thailand

## Abstract

**Background and Aim:**

Administration is the main strategy for controlling ectoparasites in dogs. Ivermectin and fipronil are most extensively used to prevent and treat *Rhipicephalus sanguineus* sensu lato infestation in dogs in Thailand. Several researchers have reported resistance to acaricides in *R. sanguineus* s.l. globally, but documentation of acaricide resistance in the tick population in Thailand is lacking. In this study, we investigated the status of resistance to commonly used acaricides in Thailand in *R. sanguineus* s.l.

**Materials and Methods:**

Engorged brown dog tick females (10 tick populations) were field-collected directly from parasitized dogs in Maha Sarakham, Thailand, for toxicological bioassays with ivermectin and fipronil. Bioassays were performed in three replicates at 25°C–27°C and 80%–85% relative humidity under a 12-h/12-h photoperiod. The 50% of lethal concentration and its confidence intervals and the slope were estimated for each tick population using probit analysis. Resistance ratios (RRs) of field ticks were characterized based on the relative susceptible population of each acaricide.

**Results:**

Six tick populations (P1–6) were tested for resistance to ivermectin, three of which (P2–4) exhibited low-level resistance to ivermectin (RR = 2.115–2.176). Of four tick populations (P7–10) treated with fipronil, two exhibited moderate-to-severe resistance (P7 and P9, RR = 21.684 and 4.387, respectively). All tick populations deemed resistant to acaricides had a history of exposure.

**Conclusion:**

Based on RR values, four *R. sanguineus* s.l. tick populations from Maha Sarakham province were resistant to ivermectin and fipronil. To the best of our knowledge, this represents the first documentation of acaricide-resistant populations of *R. sanguineus* s.l. in Thailand, and recommendations on tick control programs must be formulated with veterinarians and pet owners to prevent the development of further resistance.

## Introduction

Strategies to control ectoparasites in dogs mainly involve acaricide administration, but its efficacy varies considerably. Ivermectin and fipronil are the most extensively used acaricides for preventing and treating *Rhipicephalus sanguineus* sensu lato tick infestation in dogs in Thailand. Ivermectin is classified as a macrocyclic lactone that interacts with the gamma-aminobutyric acid (GABA) and glutamate receptors in nerve and muscle cells in arthropods and nematodes [1, 2]. In Thailand, ivermectin is widely used to control gastrointestinal parasites and ectoparasites of livestock [3]. Moreover, ivermectin is approved for veterinary use to protect dogs and cats from heartworm disease [4]. However, ivermectin has long been commonly used off-label to control ixodid tick populations in dogs and cats in Thailand based on the experiences of small-animal clinicians without an approved dosing protocol. Thus, there are safety problems and approved dosing protocols are needed.

Fipronil is also frequently used to control ticks on companion animals in this region and owners can treat their pets because the spot-on formulation is easy to apply. It is a topical acaricide product registered for tick control in companion animals in the United States in 1996 [5], and it has been widely used in Thailand. This acaricide is a phenylpyrazole that acts on GABA-gated chloride channels and blocks the flow of chloride ions, leading to neuroexcitation [6]. Ivermectin and fipronil can kill ticks, subsequently preventing direct blood loss and reducing skin irritation and the risk of tick-borne disease in dogs. The important tick-borne pathogens in dogs include *Ehrlichia canis*, *Babesia* spp., *Hepatozoon canis*, and *Anaplasma platys*, which commonly cause canine blood parasite disease in Thailand [7–10]. Nevertheless, the failure of acaricide treatment and presence of tick-borne disease in dogs are major health problems presenting as clinical and subclinical cases in Thailand [11, 12].

One obstacle to effective tick control is acaricide resistance [13]. Early detection and monitoring of resistance are necessary to delay the onset of resistance and develop strategies for tick control. Historically, the first case of acaricide resistance in *R. sanguineus* s.l. was reported in Panama, where ticks were found to be resistant to permethrin, dichloro-diphenyl-trichloroethane, coumaphos, and amitraz [14]. Subsequently, researchers globally reported resistance in *R. sanguineus* s.l. to ivermectin, fipronil, deltamethrin, and permethrin [5, 15–18]. In Thailand, few studies have focused on acaricide efficacy in *R. sanguineus* s.l. [11], and information on acaricide resistance is lacking. Thus, monitoring the efficacy of acaricides in the field and the types of acaricides being used is necessary. This useful information will enhance effective strategies to control ectoparasites.

Therefore, this study aimed to determine the resistance of brown dog ticks to commonly used acaricides in Thailand.

## Materials and Methods

### Ethical approval

This study was approved by the Institution Animal Care and Use Committee of Mahasarakham University, Thailand (IACUC-MSU-34/2022).

### Study period and location

The study collected data from May 2022 to August 2022 in Muang district, Maha Sarakham Province, Thailand.

### Tick collection

Field-collected engorged brown dog ticks were determined for ivermectin and fipronil resistance from ten populations, including four residential homes, four animal hospitals, and two dog shelters. Engorged female ticks (approximately 8–10/dog) were directly and gently removed from 10 different locations on infested dogs with forceps, and data about acaricide usage for tick control were obtained from dog owners and recorded. Information about the population of ticks and acaricide exposure is presented in Table-1. After collection, tick samples were transported to the Veterinary Parasitology Laboratory at the Faculty of Veterinary Sciences, Mahasarakham University. Then, ticks were washed thoroughly with distilled water, dried using paper towels, and morphologically identified as *R. sanguineus* s.l. under a stereomicroscope using the taxonomic method of Walker *et al*. [19].

**Table-1:** Tick collection sources and history of acaricide exposure.

Sample ID	Source	Acaricide exposure
P1	Dog shelter	Ivermectin, flumethrin, furalaner
P2	Residence	Ivermectin, flumethrin, afoxolaner
P3	Residence	Ivermectin, flumethrin
P4	Animal Hospital	Ivermectin, afoxolaner
P5	Animal Hospital	Ivermectin
P6	Animal Hospital	None
P7	Residence	Ivermectin, fipronil
P8	Dog shelter	Ivermectin, fipronil, fluralaner
P9	Animal Hospital	Ivermectin, fipronil
P10	Residence	None

Pooled ticks in each population were kept in 9 × 9 cm^2^ plastic Petri dishes. Each dish was saved by wrapping Parafilm around the edge with air holes punched into the Parafilm for ventilation. The dishes were then immediately incubated at 25°C–27°C and 80%–85% relative humidity (RH) under a 12-h/12-h light: dark photoperiod to allow egg laying and hatching [16]. On 14–21 days of age, live larvae were used for acaricide resistance bioassays.

### Ivermectin resistance assay

Absolute ethanol was used to dilute technical-grade ivermectin (Sigma-Aldrich, St. Louis, MO, USA), and 1% of ivermectin was prepared as a stock solution. An ethanol solution containing 2% of Triton X-100, diluted at 1% in distilled water and designated 1% of Eth-TX, was used as the diluent. The ivermectin stock and Eth-TX solutions were then mixed and various working concentrations were prepared. Five doses of ivermectin in Eth-TX were prepared through 50% serial dilutions from the highest concentration of 25 ppm. The final working concentrations of 25, 12.5, 6.25, 3.125, and 1.5625 ppm were used for larval immersion tests of *R. sanguineus* s.l. ticks, and 1% of Eth-TX was used as the control solution.

One milliliter of each test concentration was aliquoted into 1.5-mL microcentrifuge tubes. Approximately 200 larvae were added to each tube using a paintbrush with three repetitions for each concentration. The tube was shaken gently and larvae were immersed for 10 min. After 10 min, 100 live larvae were obtained with a new paintbrush and allowed to dry on a piece of filter paper, which was then folded, and closed with metal clips to form a packet. The larval packets were incubated at 25°C–27°C and 80%–85% RH under a 12-h/12-h photoperiod for 24 h. The packets were then opened, and live and dead larvae were counted to assess mortality [15]. The identification of dead tick larvae was based on the absence of larval movement after stimulation by a soft paintbrush and gently breathing directly onto the larvae.

### Fipronil resistance assay

To determine the resistance of *R. sanguineus* s.l. to fipronil, the protocol was somewhat modified from that described previously by Lovis *et al*. [20] and Prullage *et al*. [21]. Initially, 25 ppm fipronil was prepared as a stock solution from 10% of fipronil (Fiproline^®^, Thainaoka Pharmaceutical, Thailand) using absolute ethanol for dilution. The top and bottom portions of Petri dishes were measured, and their areas in cm^2^ were calculated and used to prepare five final working concentrations from the 25 ppm stock solution of fipronil. The Petri dishes were carefully tilted to distribute the solution over their surfaces. The final concentrations for fipronil were 1.3, 0.33, 0.08, 0.02, and 0.005 mg/cm^2^.

The dishes were left open at room temperature (35°C–37°C) and allowed to dry for 1 h. After drying, 100 *R. sanguineus* s.l. larvae were placed in the dishes. Each dish was sealed by wrapping Parafilm around the edge with air holes punched into the Parafilm for ventilation. Three replicates were used for each concentration and the control with diluent only. Larvae were incubated at 25°C–27°C and 80%–85% RH for 24 h. Then, the dishes were opened, and live and dead larvae were counted for mortality analysis.

### Statistical analysis

Mortality data were submitted to probit analysis using IBM SPSS statistics 20.0 software (IBM Corp., Armonk, NY, USA) to estimate the 50% lethal concentration (LC_50_) with its 95% confidence interval (CI) and the slope of the regression lines for each tick population and acaricide. The difference in the response to treatment in each tick population was considered significant if the 95% CIs did not overlap. Given the unavailability of a reference susceptible strain, resistance ratios (RRs) were calculated using the formula proposed by Robertson *et al*. [22] based on the tick population with the lowest LC_50_ for each acaricide.

The field populations named P6 and P10 with the lowest LC_50_ were used to calculate RRs. The susceptibility or resistance status for each population and acaricide was categorized as follows: RR < 1.5, susceptible; RR = 1.5–2, incipient resistant; and RR > 2, resistant [23].

## Results

Ten tick populations (P1–10) were collected for ivermectin and fipronil resistance assays (Table-1). Ivermectin resistance was assessed in P1–6 and fipronil resistance was analyzed in P7–10. The results of modified larval packet tests with ivermectin for six populations of *R. sanguineus* s.l. collected from the field, including the LC_50_, 95% CI, and RR, are presented in Table-2.

**Table-2:** Results of the larval immersion tests with ivermectin against *Rhipicephalus sanguineus* s.l.

Sample	N	χ ² (df)	Slope (SE)	LC_50_ (CI 95%) (ppm)	RR
P1	1896	0.264 (1)	2.007 (0.459)	2.633 (0.883–4.091)^abc^	1.093
P2	2082	21.877 (3)	1.461 (0.150)	5.213 (0.508–19.542)^a^	2.164
P3	1813	1.239 (2)	1.630 (0.21)	5.094 (3.948–6.198)^abc^	2.115
P4	1804	3.122 (3)	3.600 (0.261)	5.241 (4.748–5.784)^ac^	2.176
P5	1193	1.754 (2)	2.920 (0.256)	3.200 (2.829–3.591)^ab^	1.328
P6	1082	2.093 (2)	0.680 (0.148)	2.409 (1.123–3.767)^ab^	-

N=Number of larvae, χ²=Chi-square, df=Degrees of freedom, SE=Standard error, LC_50_=Median lethal concentration, CI95%=Confidence interval of 95%, RR=Resistance ratios, ^a, b, c^Equal letters correspond to equal LC_50_ values according CI 95% overlap

The most susceptible tick population was P6 (LC_50_ = 2.409 ppm, 95% CI = 1.123–3.767). This population was collected from a dog brought to an animal hospital and there was no history of acaricide exposure for tick control. Therefore, this population was used as the susceptible reference strain for the RR calculation. Excluding the susceptible reference group, the lowest and highest LC_50_ values for ivermectin were 2.633 (P1) and 5.241 ppm (P4), respectively.

P1 and P5 were classified as susceptible with RRs of 1.093 and 1.328, respectively. However, the RRs P2, P3, and P4 were 2.164, 2.115, and 2.176, respectively, and they were categorized as resistant to ivermectin.

P7–10 were used to assess resistance to fipronil, a commonly used acaricide in Thailand. As presented in Table-3, the LC_50_ of all populations ranged 0.450–9.758 ppm. Only one population (P10) had no history of previous exposure to fipronil, and it was identified as the most susceptible group (LC_50_ = 0.45 ppm). Therefore, this population was used as the susceptible reference strain for fipronil assays. The other three populations had confirmed prior exposure to fipronil; of these, only P8 had incipient resistance (RR = 1.453). Meanwhile, P7 and P9 were categorized as resistant strains based on their RRs of 21.684 and 4.387, respectively.

**Table-3:** Results of the larval tarsal tests with fipronil against *Rhipicephalus sanguineus* s.l.

Sample	N	χ ² (df)	Slope (SE)	LC_50_ (CI95%) (ppm)	RR
P7	1800	1.34	0.628 (0.102)	9.758 (3.428–65.832)^cd^	21.684
P8	1386	3.277	0.662 (0.102)	0.654 (0.396–1.293)^b^	1.453
P9	1800	1.801	1.164 (0.148)	1.974 (1.277–3.765)^bc^	4.387
P10	1733	4.347	0.742 (0.75)	0.45 (0.030–0.065)^a^	-

N=Number of larvae, χ²=Chi-square, df=Degrees of freedom, SE=Standard error, LC_50_=Median lethal concentration, CI 95%=Confidence interval of 95%, RR=Resistance ratios. ^a, b, c, d^Equal letters correspond to equal LC_50_ values according CI 95% overlap

## Discussion

This study demonstrated the efficacy of ivermectin and fipronil (the most commonly used acaricides) against *R. sanguineus* s.l. collected in Maha Sarakham, Thailand. Ivermectin was previously demonstrated by Tinkruejeen *et al*. [11] to have lower efficacy against *R. sanguineus* s.l. infestation in dogs in Thailand than newer acaricides [11]. Ivermectin resistance was first documented in *R. sanguineus* s.l. in Mexico in 2016 [15]. The development of ivermectin resistance has also been documented in several tick species such as *Rhipicephalus microplus* in Brazil and Mexico [24–26], *Rhipicephalus annulatus* in Egypt [27], and *Hyalomma anatolicum* in India [28]. In this study, two tick populations were susceptible to ivermectin (RR = 1.093–1.328), and three populations had low-level ivermectin resistance (RR = 2.115–2.176), all of which had a history of prior exposure to ivermectin.

The RR of brown dog ticks to ivermectin in this study was similar to those recorded in Brazil (RR = 1.54–2.97) and India (RR = 1.16–4.79) [16, 29] but lower than those recorded in Mexico (RR ≤ 30.5) and Argentina (RR = 1–18.33) [15, 30]. These differences in resistance might be related to the frequency of acaricide exposure in each region. However, the responses to treatment represented as LC_50_ in these tick populations were not considered statistically significant because their 95% CIs overlapped. The designed ivermectin concentrations for larva immersion assays in this study were lower than those used in previous studies by Rodriguez-Vivas *et al*. [15], Becker *et al*. [16], Sunkara *et al*. [29], Daniele *et al*. [30], resulting in varying mortality rates at low concentrations. Therefore, further research should be conducted in Thailand using higher concentrations of ivermectin. These results suggest that veterinarians can use ivermectin to control tick infestation in dogs following the recommended application method and frequency to slow the development of resistance in ticks. In addition, acaricide resistance must be considered when administering ivermectin to dogs because this drug has been used widely and extensively “off-label” to control dog ticks in Thailand.

Fipronil has a long history of use as an acaricide to control fleas and ticks in companion animals, especially in Thailand, because the spray and spot-on preparation is easy to administer. The first documented case of fipronil resistance in *R. sanguineus* s.l. was reported in the United States in populations of ticks from dogs with a history to exposure to spot-on formulations of this acaricide [5]. Nevertheless, later studies did not detect fipronil resistance in Florida and California, suggesting this acaricide provides suitable tick management [18]. In Brazil, resistance to fipronil in *R. sanguineus* s.l. was confirmed in a brown dog tick population in Rio Grande do Sul state [16], but a recent report demonstrated that fipronil was effective in Goiás state [31]. This study identified two tick populations with strong resistance to fipronil (RR = 4.387–21.684). These RRs were higher than those in a previous report by Eiden *et al*. [5], Becker *et al*. [16] that detected mild (RR = 1.72–3.52) and moderate (RR = 13.83) resistance to fipronil. All resistant populations in this study were collected from dogs frequently administered spot-on preparations of fipronil. Therefore, the resistance level of these populations was elevated. According to a previous report by Klaimala *et al*. [32] concerning pesticide residues in Thailand, fipronil was widely used for home termite, ant, and cockroach control, and it was one of the most common residues on home surfaces and children’s hands in one study. The rise in product availability and lower price of generic formulations of fipronil could lead to increased pet exposure to fipronil because patent protection for this drug ended in August 2010 [5, 33].

In this study, we detected acaricide resistance in *R. sanguineus* s.l. tick populations by toxicological bioassays, and to the best of our knowledge, this is the first report of acaricide resistance in this region. Further surveys are urgently needed for ticks from dogs in other areas because we lack detailed information on the epidemiology of acaricide resistance in tick populations infesting dogs in Thailand. Early detection of resistance is necessary to avoid further selection of resistant ticks. The misuse of acaricides, such as errors in preparation and application, can lead to a failure to eliminate ticks from animals [34]. In addition, long-term use and misuse of acaricides can develop resistance in ixodid ticks [35]. The occurrence of acaricide resistance in *R. sanguineus* s.l. needs better awareness and attention. Dog owners should be educated by veterinarians and encouraged to use proper application methods for acaricides, including correct dosage frequencies, alteration of different products, and correct administration, to prevent widespread resistance in the future.

## Conclusion

In this study, toxicological bioassays with commonly used acaricides were conducted using larvae of *R. sanguineus* s.l. ticks collected in Maha Sarakham, Thailand. Our results demonstrated the presence of ivermectin- and fipronil-resistant populations in this area. The presence of acaricide resistance in *R. sanguineus* s.l. should be highlighted in this region because of the role of this tick in transmitting disease pathogens to dogs and humans. Monitoring and early detection of acaricide resistance are critically important for effective tick control and the development of strategic resistance management.

## Authors’ Contributions

AJ and BS: Designed the study. AJ, BS, NN, and PK: Coordinated sample collection and performed the experiments. AJ, BS, NN, PK and ZW: Analyzed the data. AJ: Major contributor in drafting the manuscript. AJ and ZW: Revised and edited the manuscript. All authors have read, reviewed, and approved the final manuscript.
